# Are pediatric nurses prepared to respond to monkeypox outbreak?

**DOI:** 10.1371/journal.pone.0300225

**Published:** 2024-04-11

**Authors:** Bothayna Nader Sadek, Abdelaziz Hendy, Fahad M. Alhowaymel, Abdulaziz F. Abaoud, Atallah Alenezi, Ahmed Hendy, Eman A. Ali

**Affiliations:** 1 Pediatric Nursing Department, Faculty Nursing Ain Shams University, Cairo, Egypt; 2 Department of Nursing, College of Applied Medical Sciences, Shaqra University, Shaqra, Saudi Arabia; 3 Department of Computational Mathematics and Computer Science, Institute of Natural Sciences and Mathematics, Ural Federal University, Yekaterinburg, Russian Federation; SKUMS: Shahrekord University of Medical Science, ISLAMIC REPUBLIC OF IRAN

## Abstract

**Background:**

Emphasizing the crucial significance of maintaining a national nursing workforce well-prepared with the necessary knowledge, skills, and abilities to respond effectively is the growing frequency of natural and environmental disasters, coupled with public health emergencies such as the COVID-19 pandemic. So, the study aimed to explore pediatric nurses’ preparedness to monkeypox outbreak, and their stress during this outbreak in Egypt.

**Methods:**

A cross-sectional study was conducted on a 416 nurses direct care for children at selected governmental hospitals in Egypt. Demographic form, Questionnaire for Infectious Disease Outbreak Readiness & Preparedness, factors affecting nurses’ preparedness, and the generalized anxiety disorders scale-7 were the tools of the study.

**Results:**

(81.5%) of studied nurses had unsatisfactory level of preparedness to monkeypox outbreak. (96.4%) and (95.4%) of them were affected their preparedness by high workload and inconsistent income with the of risk of infection factors. Also, (57.2%) of them had high stress level.

**Conclusions:**

The study revealed the importance of ensuring adequate supplies of PPE are available and provided, and protocols must be implemented to ensure availability in case of an outbreak. Moreover, nurse staffing levels and workload distribution should be regularly reviewed to create reasonable nurse-patient ratios.

## Introduction

Monkeypox is an emerging global health threat, with increasing cases now being reported worldwide [1]. Monkeypox is an infectious disease caused by the monkeypox virus [2]. In Denmark (1958), The monkeypox virus was discovered in monkeys, and the first reported human case was in the Congo (DRC, 1970). Monkeypox steadily emerged in central, east, and west Africa. Monkeypox outbreaks appeared suddenly and rapidly spread. In May 2022, a total of 87 thousand cases and 112 deaths were reported across the Americas, Europe, and all six WHO regions, spanning 110 countries [3]. On July 23, 2022, the Director-General of the World Health Organization (WHO) declared the escalating monkeypox outbreak a Public Health Emergency of International Concern (PHEIC), which represents WHO’s highest level of alarm under international law [1].

The virus is transmissible between animals and humans, which can occur via direct contact with the lesions or bodily fluids [4]. The monkeypox virus is large in size, making puncturing the host defenses, quick replication, and evading the immune response harder [5]. Evading host immune systems occurs through replication; the monkeypox and some other orthopox viruses have evolved mechanisms to breach host immune cells [6]. Common symptoms of monkeypox include skin rash, mucosal lesions that can last 2–4 weeks, accompanied by headache, fever, back pain, muscle aches, low energy, and swollen lymph nodes [7]. The most effective methods for preventing the spread of monkeypox virus include early identification, increasing awareness regarding risk factors, and educating individuals on measures that can lead to reducing contact with the virus [8]. Young children are at high risk for severe monkeypox disease [9].

In the face of diverse public health emergencies, infectious diseases pose significant threats to the general public [10]. Active participation of healthcare workers is crucial for developing strategic plans to effectively address potential public health crises [11]. Notably, nurses play a pivotal role as the primary workforce in responding to public health emergencies, including epidemics. Their involvement in disease prevention, surveillance, and case management is paramount across various clinical settings [8]. The escalating frequency of natural and environmental disasters, coupled with the challenges posed by public health emergencies such as the COVID-19 pandemic, underscores the vital importance of having a national nursing workforce equipped with the knowledge, skills, and abilities to respond adequately. The optimization of nursing readiness is a key factor in bolstering healthcare resilience when confronted with emerging risks [12].

To effectively handle monkeypox cases, it is recommended to establish a dedicated management team consisting of a blend of enthusiastic young professionals and seasoned experts with prior experience in managing epidemics and pandemics of highly infectious diseases, such as H1N1 influenza, SARS, Ebola, COVID-19, among others. This interdisciplinary team should encompass a diverse range of healthcare professionals, including nurses, physicians, and other hospital Health Care Workers (HCWs) [13].

Offering sufficient training and education to healthcare workers (HCWs) on pandemic preparedness is crucial for enhancing their experience, knowledge, skills, and overall mental well-being during such challenging periods [14]. Additionally, gauging nurses’ current knowledge, perceptions, and training needs related to monkeypox is essential to mount an informed public health response. Any readiness gaps that exist may compromise the early detection and care for pediatric monkeypox cases. Understanding factors that may influence nurses’ outbreak preparedness can also help improve their preparedness [15].

According to cross sectional study at Saudi Arabia by Sobaikhi et al. [16] who stated that studied health care worker had low knowledge about Monkeypox. According to a national cross-sectional survey conducted by Ajman et al. [17], about half of the Health Care Workers (HCWs) included in the study expressed more significant concerns about Monkeypox disease compared to COVID-19. This heightened concern was particularly notable in relation to the potential progression of Monkeypox into a new pandemic. Additionally, a recent study by Temsah et al. [18] found that approximately 62% of the general population shared greater apprehension about Monkeypox Virus (MPXV) in comparison to COVID-19.

Nurses have a role to play in identifying patients who have been infected, ensuring proper testing, providing care, and advocating to prevent stigmatization [19]. The nursing management principles of monkeypox pediatric patients should include child isolation and protection of mucous membranes and compromised skin. Due to the loss of appetite and dehydration in the infected child, the nurse is responsible for rehydrating and providing nutritional support, checking vital signs, closely monitoring, and observing for complications [20]. Additionally, nurses must employ the highest level of personal protective equipment (PPE) when caring for children with suspected or confirmed monkeypox [21]. According to the aforementioned roles and responsibilities of nurses during monkeypox, and because of the lack of information regarding nurses’ preparedness for monkeypox, specifically for those who provide care to children, this study aims to explore pediatric nurses’ preparedness for a monkeypox outbreak and their stress during this outbreak in Egypt. Additionally, the study aims to examine pediatric nurses’ level of stress while taking care of children during a monkeypox outbreak.

The overarching aim of this study is to investigate the preparedness of pediatric nurses in Egypt to respond effectively to a potential Monkeypox outbreak. This research seeks to assess the stress, and readiness of pediatric nurses in handling Monkeypox cases, examining their level of preparedness through a comprehensive exploration of training, protocols, and available resources. The aim of this study, outlined through specific objectives, is to investigate the psychological impact of a Monkeypox outbreak on pediatric nurses, including stress levels and to assess the factors affecting their readiness and preparedness to monkeypox outbreak.

## Materials and methods

### Study design and setting

A cross-sectional research design was conducted on a convenient sample of 416 nurses to collect information about their preparedness to monkeypox outbreak. Our study was conducted over four months, from August 2023 to November 2023. The participated nurses were those provide direct care for children at selected governmental hospitals affiliated to Egyptian Ministry of Health and Population and university hospitals. A total of ten hospitals were surveyed in this stud; they were located in different regions of Egypt. Three hospitals were from the north region, three hospitals were from the south region, and four hospitals were from the middle region of the country.

### Study population

The study included 416 pediatric nurses selected through a convenience sample. Inclusion criteria for this study encompassed nurses with a minimum of one year of previous work experience, actively practicing during the COVID-19 quarantine, providing direct care to children, still actively practicing nursing, and willing to participate in the study. Those who did not have at least one year of experience or were no longer providing nursing care to children were excluded from the study.

### Sample size

Calculate sample size for study dependent on results of Hendy et al. [22] who revealed that mean score of nurses inasequate preparation was 9.92 (SD 2.436) and mean score of total nursing stress scale was 99.47 (SD 10.671), with confidence level 99% and margin of error 0.32

By using the sample size formula:

n = (Z-score)2 * SD2 / MOE2

Where:

Z-score for 99% confidence = 2.58, SD = 2.436, MOE = 0.32

n = (2.58)2 x (2.436)2 / (0.32)2

n = 6.67 x 5.93 / 0.1024

n = 374

Therefore, with 99% confidence and a margin of error of 0.32, the required sample size for the study was at least 374 pediatric nurses and adding attrition with 42, so the final sample size was 416.

### Data collection tools

#### Demographic information

The demographic questions in the study were about age, gender, level of educational, experience, marital status, workplace, working hours Additional questions were also added to ask about nurses’ level of awareness about current situation of infectious disease outbreaks, and recent guidelines of preparedness of monkeypox outbreak, and if nurses have received training about their role in controlling monkeypox outbreak in children. Additional eight questions about factors that may influence nurses’ preparedness to monkeypox outbreak were add to the demographic questions (e.g., high workload and insufficient training).

#### The Staff Questionnaire for Infectious Disease Outbreak Readiness & Preparedness (SQIDORP)

The SQIDORP was adapted from Jokwiro et al. [23] to assess nurses’ preparedness to infectious diseases outbreak and composed of 19 items: these items were distributed into 4 categories, including perceived training (3 items), hospital administrative support (4 items), nurses’ preparedness to monkeypox outbreak (9 items) and perceived support (3 items). A 3-point Likert scale was used ranged from (1 = not agree to 3 = agree). Scores ranged from 19 to 57 with high scores indicating a satisfactory level of preparedness and categorized as follow: satisfactory level of preparedness 39–57, and unsatisfactory level of preparedness 19–38

#### The Generalized Anxiety Disorder Scale-7 (GAD-7)

The GAD-7 was developed by Spitzer et al. [24]. The scale consisted of 7 self-reported items described the most salient feelings of GAD-7 including “feeling nervous, anxious or on edge, not being able to stop or control worrying, worrying too much about different things, trouble relaxing, being so restless that it is hard to sit still, becoming easily annoyed or irritable, and feeling afraid as if something awful might happen”. A 4-point Likert scale was used ranged from (0 = not at all to 3 = nearly every day). Scores above 10 are considered to be in the clinical stress range [24]. Scores ranged from 0 to 21 with high scores indicating severe anxiety symptoms and categorized as following: 0–7 low stress, 8–14 moderate stress and 15–21 high stress.

### Validity and reliability of the data collected

A language expert translated the measures into Arabic for this study, and experts in pediatric and critical care nursing assessed the face and content validity. The experts assessed the measures design, content, consistency, relevancy, and accuracy of the tools. In addition, measure reliability was assessed using the Cronbach alpha coefficient statistical test. The SQIDORP and GAD-7 had adequate internal consistency reliability of 0.869 and 0.911, respectively.

### Data collection

In order to reach out to eligible participants, the research team reached out first to the head nurses of pediatric units at the surveyed hospitals. The team explain the study, its aims, and our targeted nurses to head nurses. Then, the team has shared an online prepared questionnaire with head nurses to be shared with pediatric nurses working in their units. the online questioner included measures mentioned above, and it was restricted be filled out one time by each participant. The questionnaire began with the consent includes purpose and process of the study, and selection options of agree, or refuse to participate in the study. After the nurse agreement of consent form, the nurse clicks “Next” for beginning to answer the questions. The follow up procedure was done with head nurses.

### Ethical considerations

Before collecting data, the research team obtained an ethical approval from the Ethical Committee of the Faculty of Nursing Ain Shams University **(ID#: 22.10.36)**. The participated nurses were informed about the purpose of the study and the written approval was obtained from each nurse before participating at study, the study was conducted from 1/8/2023 to 20/102023. They were also informed that their participation in the study is voluntary, and they have the right to withdraw from the study at any time. For the privacy and anonymity of data, the data were deidentified. The data collected for the study was stored securely with password.

### Statistical analysis

The Statistical Package for Social Sciences (IBM SPSS) version 26 use used for data analysis for this study. Descriptive analyses including numbers, percentages, and means were reported for study variables. Inferential analyses were also applied in this study. A statistical model of multiple linear regression was used to predict factors affecting the study variables with dependent continuous variable (Preparedness scale). An odds ratio (OR) was also used to measure the quantifies the strength of the association between variables.

## Results

### Characteristics of participants

Table 1 revealed that, 218 (52.4%) of nurses aged between 30 to <40 years with the mean age of them was 33.17 (SD 4.99) years. They were 277 (66.6%) females and 139 (33.4%) were males. Out of 416 nurses, 189 (45.5%) had more than 15 years of experience (mean 12.01; SD 3.25). Moreover, 215 (45.5%) of nurses worked 12 hours and 118 (28.4%) worked part time. Also, 394 (94.7) didn’t receive any training courses about monkeypox, 324 (77.9%) and 320 (76.9%) were aware of the current situation of monkeypox outbreak and world health organization guidelines, respectively.

**Table 1:** Distribution of studied nurses according to their characteristics (n = 416).

Items	n	%
Age:		
20 - <30	141	33.9
30 - <40	218	52.4
40–50	51	12.3
>50	6	1.4
Mean ± SD 33.17±4.99		
Education level:		
Diploma of nursing	37	8.9
Technical health institute	181	43.5
Bachelor of nursing	164	39.4
Postgraduate	34	8.2
Years of experience:		
1 - <5	73	17.5
5 - <10	64	15.4
10 –<15	90	21.6
15 year or more	189	45.5
Mean ± SD 12.01±3.25		
Marital status:		
Married	288	69.2
Unmarried	128	30.8
Gender:		
Males	139	33.4
Females	277	66.6
Job description		
Head nurse	231	55.5
Staff nurse	185	45.5
Working status		
Full time	298	71.6
Part time	118	28.4
Working hour		
6	179	43
12	215	51.7
24	22	5.3
Attended training courses about monkeypox		
Yes	22	5.3
No	394	94.7
Aware with current situation of monkeypox outbreak		
Yes	324	77.9
No	92	22.1
Aware with WHO guidelines of monkeypox outbreak		
Yes	320	76.9
No	96	23.1
If yes, source of information (n = 320)		
Hospital	109	34.1
Social media	115	35.9
Browsing	96	30

### Preparedness and stress level among studied nurses

**Fig 1:**
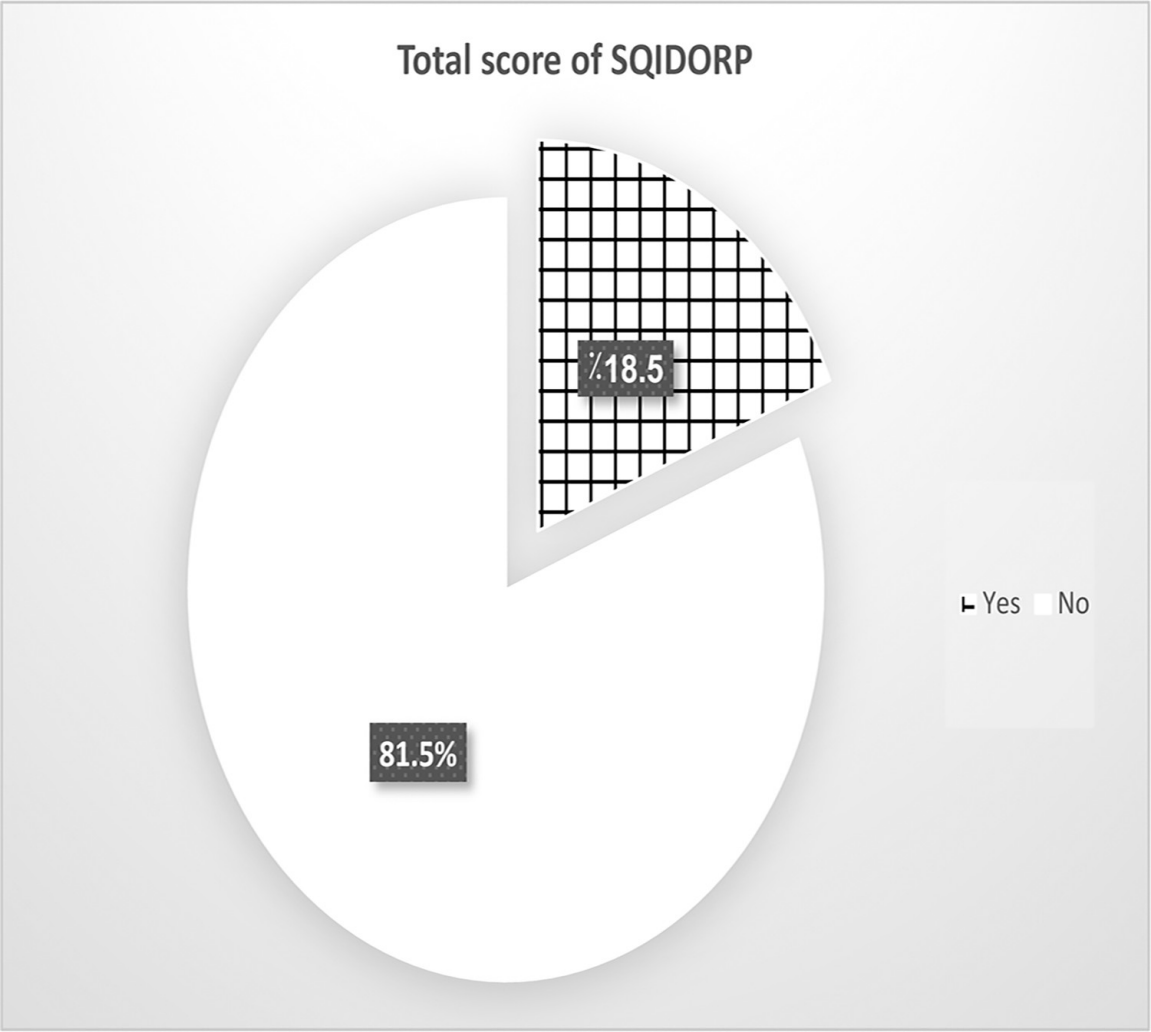
Percentage distribution of studied nurses according to their total scores of SQIDORP questionnaire (n = 416).

**Fig 2:**
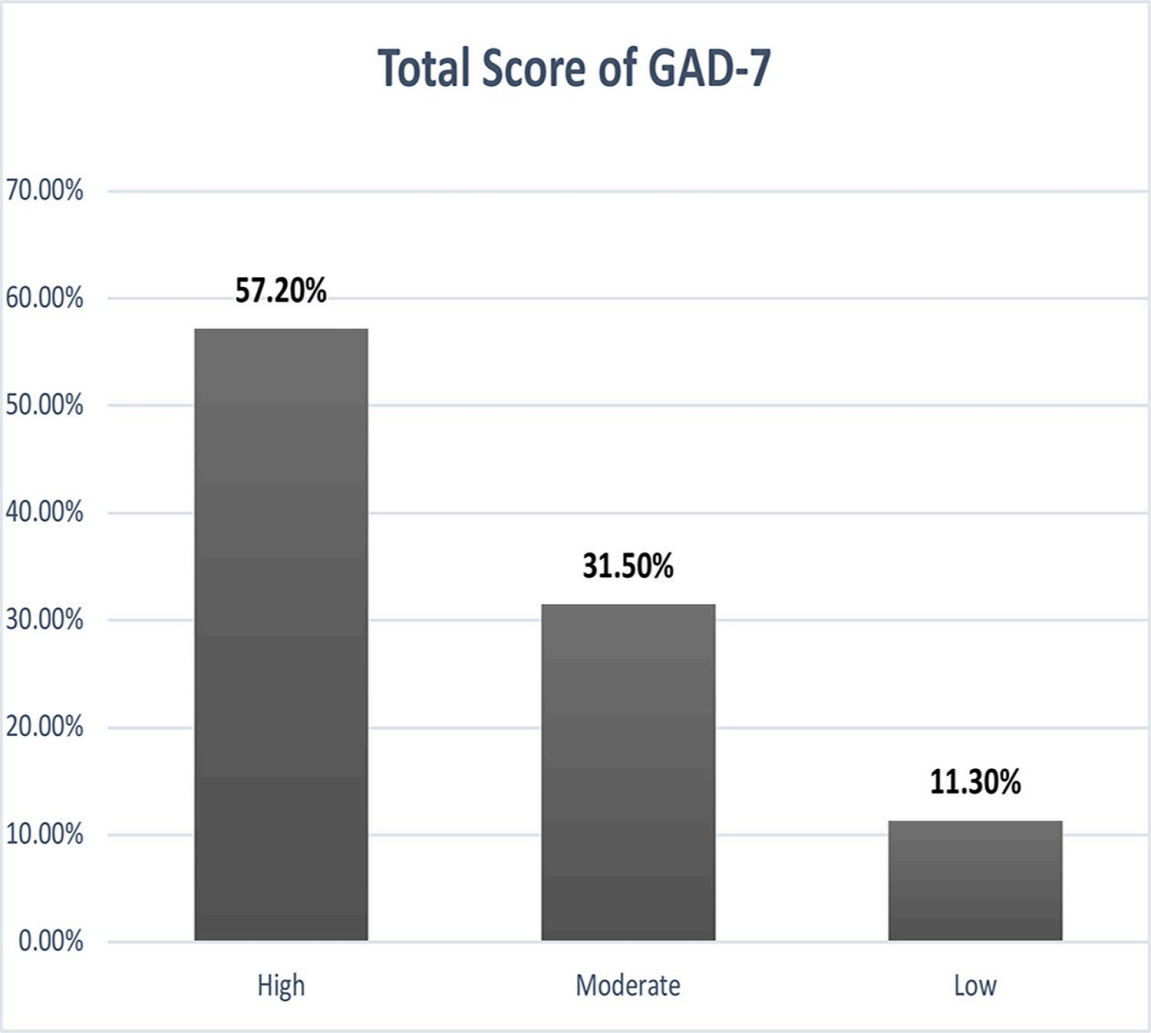
Distribution of studied nurses according to their total score of GAD-7 (n = 416).

The reported mean score of the SQIDORP was 23.43 (SD 3.70) which indicated unsatisfactory preparedness among pediatric nurses. Only 77(18.5%) of the studied nurses had satisfactory level of preparedness to monkeypox outbreak (Fig 1). High workload and inconsistent income with the risk of infection were the most two reported factors by nurses that affect their preparedness to monkeypox outbreak (Table 2). In terms of stress, the mean score of stress reported by pediatric nurses was 14.87 (SD 2.89) that indicate relatively high stress (Fig 2). 131 (31.5%) of nurses reported moderate level of stress, 238 (57.2%) had high level, whilst 47(11.3%) of them had low level of stress.

**Table 2:** Distribution of studied nurses according to factors affecting their readiness and preparedness to monkeypox outbreak (n = 416).

Factors	n	%
High workload	401	96.4
Annoyed by the increased number of working hours	353	84.9
Unavailability of protective measures	199	47.8
Unavailability of disinfectants solutions	181	43.5
Income is inconsistent with the of risk of infection	397	95.4
Lack of support	246	59.1
Exposed to relative violence	101	24.3
Insufficient training	210	50.5

### Factors affecting preparedness level

The statistical model of multiple linear regression explains 58.5% of the variance, indicating moderate predictive ability. Nurses stress negatively predicted their readiness and preparedness (*p* = .007). For every unit increase in stress, there is a decrease by 1.90 in nurses’ preparedness. Among other included factors, nurses’ educational level (*p* = .009); awareness of WHO guidelines (*p* = .008); and perceived training program (*p* = 0.043) positively predicted nurses’ preparedness. However, working hours (*p* = 0.015) and previous experience (*p* = .034) negatively predicted nurses’ preparedness (Table 3).

**Table 3:** Multiple linear regression model associated of nurses’ preparedness to monkeypox outbreak (n = 416).

Model	Unstandardized B	Coefficient St. Error	Standardized Coefficient Beta	t	sig
(Constant)	13.231	2.42		10.410	.001
Stress	-1.901	.841	-.379	-5.756	.007*
Educational level	1.243	.611	.210	4.661	.009*
Working hour	-0.976	.412	-.196	3.014	.015*
Previous experience	-0.933	.387	-.170	2.915	.034*
Aware with WHO guidelines	1.301	.700	.286	5.001	.008*
Perceived training program	0.831	.302	.142	2.665	.043*
Model summary					
R	R square		F	sig	
.765	.585		12.513	.000	

Dependent variable: Nurses’ preparedness

Predictors: (constant), Stress, Educational level, Working hour, previous experience, Aware with WHO guidelines, and Perceived training

The results in Table 4 shows the association between the possible predicted factors and nurses’ preparedness. Nurses who suffer from high workload are four times (4.8) more prone to unpreparedness than others, with high significant relation at p value .003. Also, unavailability of protective measures are four times (4.21) more negatively affected their preparedness to monkeypox outbreak than others with high significant relation at *p* = .002. In addition, Lack of training are three times (3.33) with *p* = 0.029 (Table 4).

**Table 4:** Odds ratios for association between predicted factors and unsatisfactory preparedness.

	Odd Ratio	95% CI		Chi-square	P
		Low	High		
High workload	4.8	1.23	17.65	8.824	.003
annoyed by the increased number of working hours	2.01	1.09	7.530	5.002	.019
Un availability of protective measures	4.21	1.16	15.63	9.80	.002
Availability of disinfectants and sterilization solutions	2.98	1.03	10.81	5.007	.016
Income is inconsistent with the level of risk of infection	0.021	0.009	1.557	1.222	.076
Lack of appreciation during working at COVID-19 pandemic	2.00	1.32	13.62	6.002	.008
Exposed to relative violence	2.62	1.04	6.585	4.492	.035
Lack of training	3.33	.906	12.26	4.791	.029

## Discussions

Monkeypox outbreak has become a new global challenge after COVID-19 pandemic. The spread of prevailing outbreaks has a huge burden on healthcare systems and focused on the governmental decision for enabling the healthcare systems to respond and fight against prevailing outbreaks. Accordingly, a disaster plans were established for hospital preparedness as well as healthcare personnel especially frontline nurses [25]. The negative effect of work stress has been significant on their preparedness to infectious outbreaks [26]. However, the frontline nurses’ preparedness for responding to infectious outbreaks has not been sufficiently understood [27]. Moreover, most studies focused on the organizational abilities during outbreaks, while attention paid to the role of frontline nurses is too little in controlling infectious outbreaks [28]. There is lack of information identified in the literature for explaining the role of pediatric nurse in preparedness during monkeypox outbreak.

The current findings prove that most of nurses had unsatisfactory levels of preparedness for monkeypox outbreak. Therefore, investigating the factors affecting nurses’ preparedness is paramount to respond to monkeypox outbreak. the current study revealed that most observed factors affecting the unsatisfactory preparedness as reported by the studied nurses were high workload, inconsistent income with the risk of infection and annoyed by the increased number of working hours, lack of support, and insufficient training. These findings are consistent of Hessels et al. [29] who mentioned that most of nurses reporting an extra-workload burden and a lot of time spent in isolation precautions. The current results were contradictory to the study done by Awamleh [30] who reported that three quarters of nurses perceived themselves to be well-prepared to covid-19 pandemic. A possible explanation to our findings is that most of nurses included in the current study were working more than 12 hours and reported that their income is not proportional to the workload and risk of infection.

The present study revealed that more than half of the surveyed nurses experienced a high level of stress. Additionally, over one-quarter of them reported moderate stress levels, a phenomenon possibly linked to prior encounters with heavy workloads, fear of infection, and the social stigma surrounding the COVID-19 pandemic. These findings align with the study conducted by Hendy et al. [31], which indicated that half of the nurses under investigation reported moderate stress levels, and a quarter of them experienced severe stress. Similarly, Jiang’s study [32] highlighted that hospital staff faced work-related stress during the COVID-19 outbreak. Moreover, ÇELİK et al. [33] found that nearly half of the participants lacked comprehensive knowledge about the disease before the surge in monkeypox cases, leading to varying levels of depression, anxiety, and stress regarding a potential new pandemic.

Our study results suggest that educational level, awareness of WHO guidelines, and training program were the positive predictive factors of overall preparedness. Other studies highlighted knowledge, experiences, and practice improved nurses’ readiness through training prior to the outbreak influenced preparedness in addition to nurses’ awareness of their role during the infectious outbreak (Fernandez et al. [34]). The enrolled nurses in our study had working experience during COVID-19 pandemic which considered an important issue in acquiring knowledge and training related to infection control safety measures. Strengthening training, preparedness programs awareness of recent guidelines are effective methods to alleviate stress among nurses (Grace & VanHeuvelen [35]). Also, Mahanta et al. [36] reported that the practice, knowledge, and attitude, (KAP) of HCWs toward the COVID-19 pandemic influence their preparedness.

Previous studies on nurses’ preparedness have shading lights on the importance of training for support frontline nurses’ willingness to prepared to fight infectious epidemics, but most of nurses always feel that they have not received enough training (Adongo et al. [37]; Almutairi et al. [38]; Chen et al. [39].

In this study, work stress, working hours, and previous experience were negative predictor factors affecting preparedness to monkeypox outbreak among studied nurses. Also, high workload and unavailability of protective measures were four times negatively affected nurses’ preparedness for monkeypox outbreak which may be due to limited nurses’ knowledge and skills regarding monkeypox in children, methods of transmission, preventive & protective measures against monkeypox outbreak. These results were consistent with the study done by Kim & Choi [40] who reported that stress of workload, poor hospital resources, poor protective measures, and poor support from family and friends were negative predictor factors of stress among nurses.

In our study nurses’ preparedness was negatively correlated with stress. Relevant studies reported that readiness of nurses when dealing with an infectious outbreak reflected varying degree of psychological problems, including stress, worry, fear from infection, and health concern (Adongo et al. [37]; Chen et al. [39]; Fryk et al. [41]. Also, Disaster preparedness was negatively related with mental health, including depression and anxiety Ying et al. [42].

### Limitations of the study

While conducting the study, we encountered some limitations. The study relied on a convenience sample of pediatric nurses from selected governmental hospitals in Egypt. This may introduce selection bias, as nurses who volunteered to participate may differ from those who did not, affecting the external validity of the results. Additionally, the study depended on self-reported data from nurses, including their preparedness levels and stress. This introduces the possibility of response bias.

### Conclusions

The majority of nurses had unsatisfactory levels of readiness and preparedness for a potential monkeypox outbreak. Key factors negatively affecting readiness identified were high workload, insufficient income versus infection risk, and higher stress levels. Increased education, WHO guideline awareness, and perceived quality of training programs positively predicted preparedness, while more working hours and lack of previous outbreak experience were detrimental. Healthcare systems and legislators should take into consideration specific factors that may influence nurses’ preparedness to emergencies and epidemics such as Monkeypox. For example, adequate supplies of PPE should be available and provided, and protocols must be implemented to ensure availability in case of an outbreak. Moreover, nurse staffing levels and workload distribution should be regularly reviewed to create reasonable nurse-patient ratios. Finally, a monkeypox-specific training program are highly encouraged to be developed and validated for nurses that can include strategies for coping with stressful outbreak responses.

## Supporting information

S1 Dataset (XLSX)
